# Pertussis Toxin B-Pentamer Mediates Intercellular Transfer of Membrane Proteins and Lipids

**DOI:** 10.1371/journal.pone.0072885

**Published:** 2013-09-03

**Authors:** Scott H. Millen, Olivia D. Schneider, William E. Miller, John J. Monaco, Alison A. Weiss

**Affiliations:** Department of Molecular Genetics, Biochemistry, and Microbiology, University of Cincinnati College of Medicine, Cincinnati, Ohio, United States of America; Ecole Polytechnique Federale de Lausanne, Switzerland

## Abstract

Pertussis toxin (PTx) is the major virulence factor of *Bordetella pertussis*. The enzymatic or active (A) subunit inactivates host G protein coupled receptor (GPCR) signaling pathways. The non-enzymatic binding (B) subunit also mediates biological effects due to lectin-like binding characteristics, including the induction of T cell receptor (TCR) signaling and subsequent down-regulation of chemokine receptor expression. Here we report another activity attributable to PTxB, facilitating transfer of membrane material between mammalian cells. This activity does not require the TCR, and does not require cell-to-cell contact or cellular aggregation. Rather, membrane vesicles are transferred from donor to recipient cells in a toxin-dependent fashion. Membrane transfer occurs in different cell types, including cultured human T cells, CHO cells, and human primary peripheral blood mononuclear cells. Transfer involves both lipid and integral membrane proteins, as evidenced by the transfer of T and B cell-specific receptor molecules to other PBMCs. Interestingly, membrane transfer activity is a property that PTx shares with some, but not all, cell-aggregating lectins that are mitogenic for human T cells, and appears to be related to the ability to bind certain host cell glycolipids. This phenomenon may represent another mechanism by which pertussis toxin disrupts mammalian intra- and inter-cellular signaling.

## Introduction

Whooping cough, or pertussis, is a highly contagious disease caused by the Gram-negative bacterium, *Bordetella pertussis*. For the last two decades, the number of cases of pertussis in the United States has been increasing even though vaccination rates have remained very high, and poor efficacy of the new acellular vaccines has been proposed as an explanation for the rising disease rate. Pertussis toxin (PTx) is arguably the most important virulence factor of *B. pertussis,* and the most critical vaccine antigen for the prevention of serious, life-threatening disease. PTx is a member of the AB_5_ family of bacterial toxins. The single enzymatically active (or A) subunit (called S1) is an ADP-ribosyltransferase, an enzyme that inactivates the α-subunit of some GTP-binding proteins [1]. The binding (or B) subunit is a pentamer composed of 4 different polypeptides, S2, S3, S4, and S5, in the ratio 1∶1∶2∶1, respectively. The B-pentamer is required for cell binding and delivery of the S1 enzyme into the mammalian cell cytoplasm.

Interestingly, the B-pentamer also mediates toxic activities independently of the A-subunit ADP-ribosyltransferase activity. B-pentamer activities include mitogenicity and T cell activation [2], [3]. The B-pentamer activates the T cell receptor (TCR) by clustering the receptor proteins in a manner similar to antibodies against CD3, a key signaling protein in the TCR complex. In contrast to anti-CD3 antibodies, the PTx B-pentamer promotes clustering by binding to the glycan residues that decorate the CD3 glycoproteins [4]. Plant lectins such concanavalin A (ConA), phytohemagglutinin leucoagglutinin (PHA-L), and wheat-germ agglutinin (WGA) also activate the TCR by binding to glycans on the TCR proteins. Glycan recognition is relatively non-specific, and in addition to promoting the receptor clustering that leads to activation of the TCR, pertussis toxin also promotes cellular aggregation.

We began this study intending to examine PTx-mediated cellular aggregation. To do this, Jurkat T cells were stained separately with two lipophilic fluorescent dyes, DiO (Green) or DiD (Red), mixed together in the presence of PTx, and analyzed by flow cytometry. As expected, a ‘double positive’ population consisting of clusters containing both red and green cells was observed. However, an unexpected population was also seen. Some individual cells strongly stained for one dye displayed a light staining with the other dye. We demonstrated that this staining was due to transfer of subcellular membrane vesicles onto intact cells. Membrane transfer occurred in other cell lines and, importantly, was also seen using cells derived from the blood of human donors. Ptx was required for the transfer of membrane to the recipient cell, but not for the generation of the vesicles that are transferred. This membrane transfer also moves membrane-associated cell-surface signaling proteins between cells in a nonspecific manner. For example, PTx-treatment caused T cell receptor (CD3) to be acquired by human B cells and monocytes. The ability of a pertussis toxin to scramble the markers displayed on immune effector cells could have important implications in the disease process, as well as altering the ability to promote long-term protection from infection when used as a vaccine antigen.

## Results and Discussion

### Ptx B-pentamer Promotes Cellular Aggregation and Membrane Transfer between Cells

**Figure 1:**
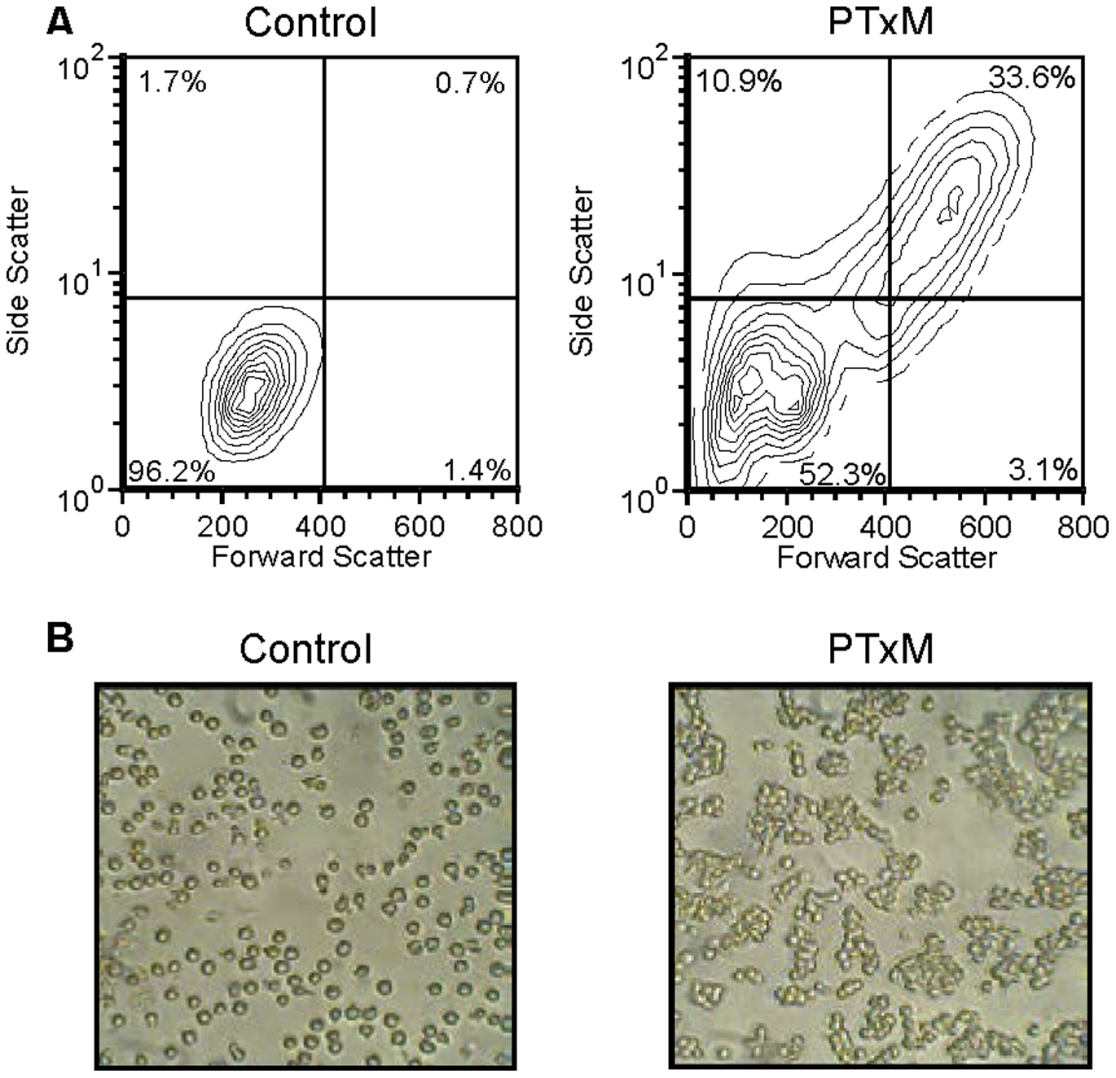
PTxM-mediated aggregation of Jurkat cells. A. Analysis of by flow cytometry showing the forward scatter and side scatter profiles. Control, untreated cells (1 hr at 37°C); PTxM treated (7.9 nM for 1 hr at 37°C). B. Microscopic examination of Jurkat cells, control and PTxM treated as described above.

To study the effects of the B-pentamer lectin activity in the absence of the A-subunit ADP-ribosylation activity, throughout this study we used the genetically toxoided form of pertussis toxin, PTxM. PtxM contains the normal complement of wild type B subunit polypeptides, but harbors a single amino acid substitution in the A subunit that abrogates its enzymatic activity. Human Jurkat T cells were treated with PTxM for 1 hr at 37°C and analyzed by flow cytometry. PTxM treatment induced a change in the forward and side scatter profiles of Jurkat cells (Fig. 1A). Microscopic examination revealed the formation of cell aggregates (Fig. 1B). The larger and more complex population seen by flow cytometry is likely due to a multivalent agglutination activity similar to the previously reported hemagglutination activity of PTxB [5]–[7].

Initially, a decrease in event rate was observed by flow cytometry for the PTxM treated cells compared to untreated cells. Cellular loss was not observed in the microscopic images, suggesting formation of aggregates too large to be detected by flow cytometry. In subsequent studies, samples for flow cytometry were mixed by vigorous pipetting. This resulted in a higher event rate, and indicates that residual aggregates detected by flow cytometry represent tightly associated cells.

**Figure 2:**
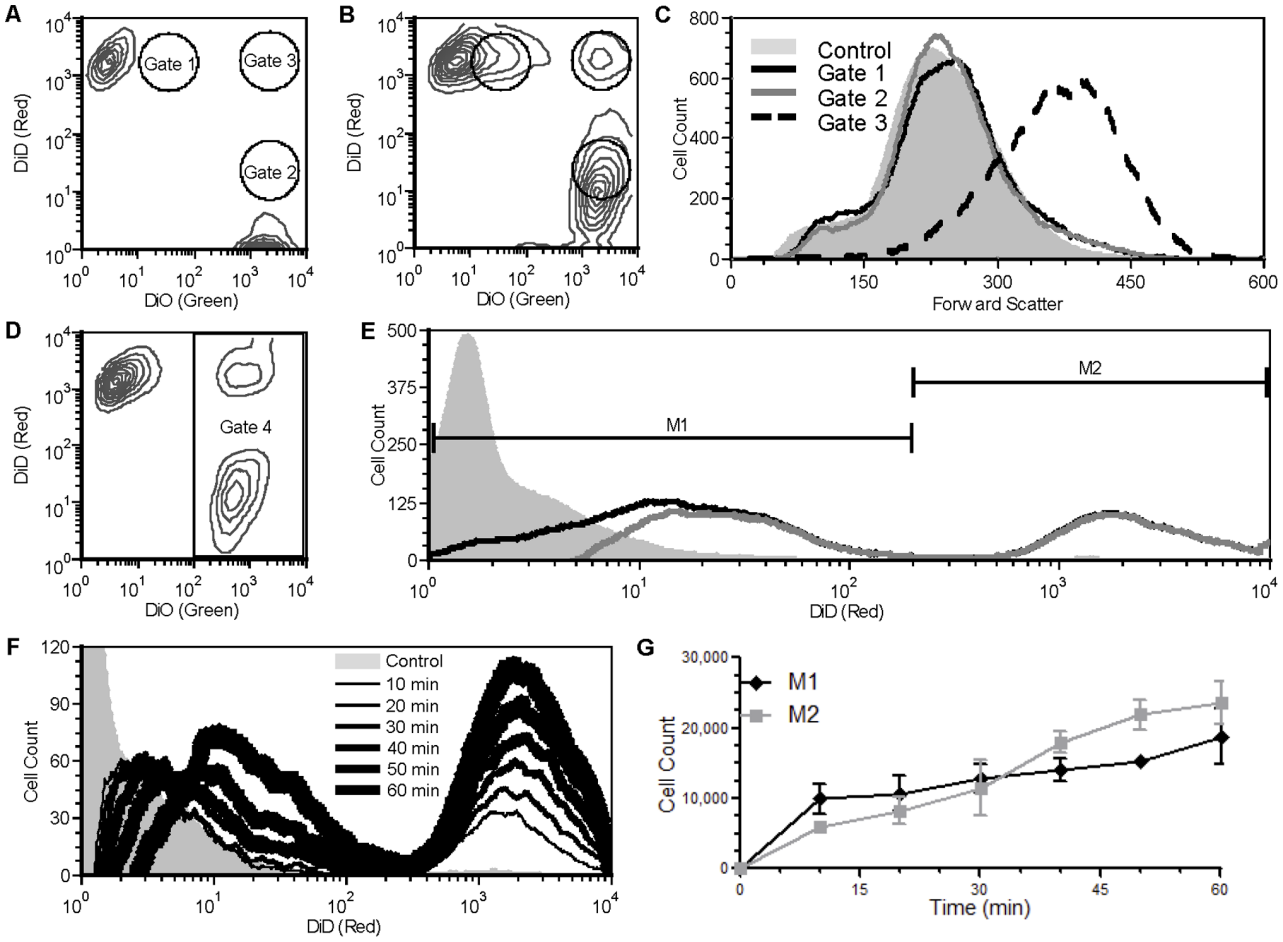
Flow cytometry analysis of stained Jurkat cells. Analysis by flow cytometry of mixed DiO (10 µM) and DiD (5 µM) stained Jurkat cells, showing the green fluorescent/DiO and red fluorescent/DiD profiles with relative positions of gates 1, 2, & 3: **A**. Untreated (1 hr at 37°C). **B**. PTxM treated (7.9 nM for 1 hr at 37°C). **C.** Analysis of cells showing the forward scatter profiles of the ungated cells in panel A (control) and gated cells in panel B. **D.** Mixed DiO- (5 µM) and DiD- (5 µM) stained PTxM-treated cells showing the green fluorescent/DiO and red fluorescent/DiD profiles and the position of gate 4. **E.** Red fluorescent/DiD profiles of untreated (grey field) and PTxM-treated (black line) Jurkat cells from Gate 4, and Overton subtraction of untreated from treated (grey line). Transfer (M1) and aggregation (M2) events are indicated. **F.** Analysis by flow cytometry of Jurkat cells showing the red fluorescent/DiD profiles of the Gate 4 cells with no treatment (grey field) and a time course for PTxM treatment from 10 min (thinnest black line) to 60 min (thickest black line). **G.** Comparison of the mean transfer (M1, black line) and aggregation (M2, grey line) population sizes of three independent assays with standard deviation.

**Figure 3:**
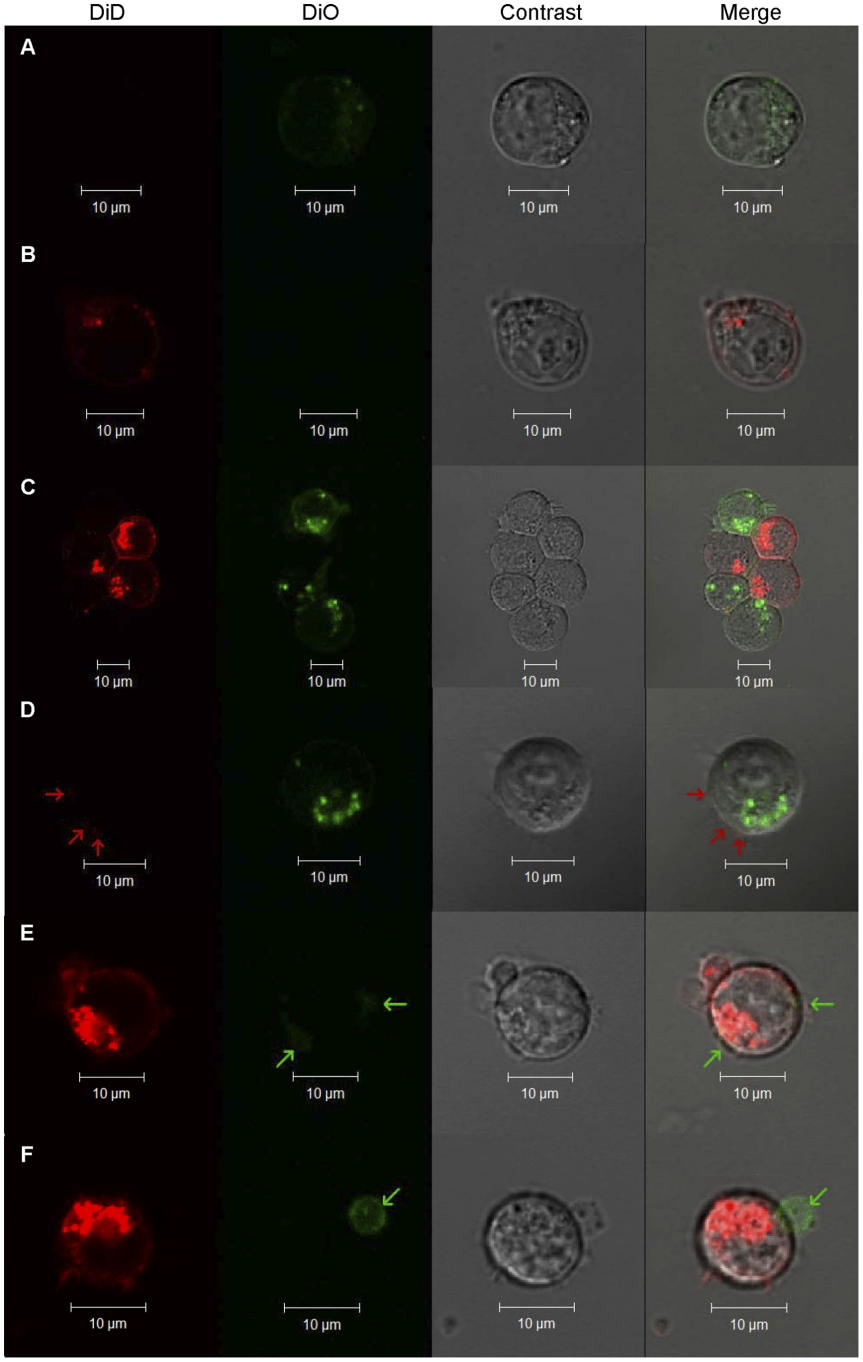
Confocal microscopy of stained Jurkat cells. Representative confocal microscopy images, showing the DiD channel, the DiO channel, differential interference contrast, and a merged image, of mixed DiO (5 µM) and DiD (5 µM) stained Jurkat cells treated with PTxM (7.9 nM for 1 hr at 37°C)and sorted into the three gates depicted in Figure 2A. **A.** Unsorted and untreated DiO- stained (Green) control Jurkat cells (8 µm *z*-plane). **B.** Unsorted and untreated DiD-stained (Red) control Jurkat cell (10 µm *z*-plane). **C.** Aggregation of DiO and DiD stained cells resulting from PTxM treatment sorted by gate 3 (7 µm *z*-plane). **D.** Gate 2 sorted DiO-stained cell with DiD-stained vesicles (red arrows, 6 µm *z*-plane). **E.** Gate 1 sorted DiD-stained cell with DiO-stained vesicles (green arrows, 7 µm z-plane). **F.** Gate 1 sorted DiD stained cell with DiO stained vesicle (green arrow, 9 µm z-plane).

To examine the aggregation process in more detail, a Jurkat cell population was divided into two, and one half was stained with the lipophilic green fluorescent dye DiO and the other half was stained with the lipophilic red fluorescent dye DiD; for simplicity, we will refer to these as Red and Green cells. Green and Red populations were mixed and analyzed by flow cytometry. As expected, stained but PTx-untreated (control) cells revealed two distinct populations (Green^+^/Red^−^ and Green^−/^Red^+^) (Fig. 2A), while cells treated with PTxM revealed the presence of a population of double positive (Green^+^/Red^+^) signals (Fig. 2B, coinciding with the position of gate 3). Forward scatter revealed that the Green^+^/Red^+^ signals from gate 3 (Fig. 2C, dashed lines) were larger than single cells from the untreated control (Fig. 2C, shaded histogram), and microscopic examination revealed that they were composed of aggregates of approximately two to six cells (Fig. 3C). Therefore, we will refer to the PTx-mediated appearance of the Green^+^/Red^+^ population in Gate 3 as an aggregation event.

In addition to the aggregated cells in Gate 3, we observed that some of the green-negative, red-positive cells (Green^−/^Red^+^) present in the non-PTx-treated population appeared to be shifted slightly into the green dimension after PTx treatment, resulting in an intermediate population between Green^−/^Red^+^ and Green^+^/Red^+^ (Fig. 2B, coinciding with the position of gate 1), and a similar intermediate shift to red fluorescence was observed for the Green^+^/Red^−^ population (Fig. 2B **,** coinciding with the position of gate 2). We will refer to the intermediate populations occupying Gate 1 as Green^δ+^/Red^+^, and Gate 2 as Green^+^/Red^ δ+^. Non-PTx-treated control cells still displayed single color fluorescence after mixing (i.e., all red or all green), demonstrating that the dye did not mix between cells after staining (Fig. 2A). While the green dye DiO is known to red-shift with molecular level clustering [8], the lack of red-shift in the control cells suggests this phenomenon is not responsible for the Green^+^/Red^δ+^ population seen with the PTxM-treated cells.

The signals from Gates 1 and 2 displayed forward scatter characteristics identical to the non-PTx-treated controls cells, suggesting that they represented single cells and not aggregates (Fig. 2C), and this was confirmed by microscopic examination (Fig. 3 D–F). The appearance of the single-cell populations with intermediate fluorescence profiles in Gates 1 and 2 was unexpected. Both lipophilic dyes are anchored to the plasma membrane by two 18-carbon aliphatic chains. The PTxM-dependent acquisition of dye for the population of non-aggregated cells in Gates 1 and 2 must be due to cellular acquisition of stained membrane material. We will refer to the single cell, PTxM-dependent acquisition of dye as a membrane transfer event.

Since both Red and Green populations appear to be equivalent, for simplicity we applied a gate to limit the analysis to only the Green^+^ events (Fig. 2D, Gate 4). Fig. 2E presents the Red fluorescence profile of Green^+^ cells treated with PTxM (Fig. 2E, black line), compared to non-PTx-treated control cells (Fig. 2E, light gray histogram). Subtraction of the control from the PTx-treated histograms [9], generated the histogram (Fig. 2E, dark gray line) that was used to distinguish membrane transfer events (Fig. 2E, M1) from aggregation events (Fig, 2E, M2).

A time course of PTx-B treatment reveals a rapid and time-dependent increase in both the Green^+^/Red^δ+^ and Green^+^/Red^+^ populations (Fig. 2F, the time point histograms, black lines, are Overton subtractions as described in Fig. 2E, grey line). Both populations are apparent after only 10 minutes of incubation with toxin, and the number of cells within each population continues to increase throughout the incubation period (Fig. 2G). Interestingly, the intensity of red staining in the Green^+^/Red^+^ (M2) population does not increase over time, consistent with this population representing small, stable aggregates of cells. In contrast, the intensity of red staining in the Green^+^/Red^δ+^ (M1) population increases from the 10 minute time point through the 50 minute time point, and appears to begin to plateau by about 60 min (Fig. 2F). These kinetics suggest that the two events can occur independently (i.e., aggregation is not an obligate precursor to membrane transfer, see below).

In order to visually examine the nature of the membrane transfer, PtxM treated Jurkat cells were sorted into Green^δ+^/Red^+^, Green^+^/Red^δ+^, and Green^+^/Red^+^ populations based on the gates shown in Fig. 2A and examined by confocal microscopy. Unsorted non-mixed Green^+^ and Red^+^ cells were used for control purposes. Green^+^ (Fig. 3A) and Red^+^ (Fig. 3B) control cells revealed diffuse plasma membrane staining, and punctate intracellular staining, consistent with membrane invagination, similar to staining patterns reported in earlier studies [10]. As discussed above, the Green^+^/Red^+^ sorted population revealed small clusters of 2–8 mixed Green^+^ and Red^+^ cells (Fig. 3C).

The Green^+^/Red^δ+^ sorted population revealed cells with green cytoplasmic staining, and visible red staining was only associated with the cell-surface. Frequently the red stain appeared to be present as very small extracellular spheres (1–5 µm in diameter) on the cell-surface (Fig. 3D, red arrows). While the red stain is difficult to visualize in any single confocal image, red stain was visible on multiple areas of the cell surface when the entire cell was examined, which together generated enough signal to allow the cell to be sorted into the red channel (data not shown). Similarly, the Green^δ+^/Red^+^ sorted population revealed Red^+^ cells with green-stained small extracellular spheres (Fig. 3E, green arrows). It was also common to see large extracellular spheres attached to the cell surface. A large red sphere was seen on a Red^+^ cell (Fig. 3E), and interestingly a large green extracellular sphere was seen on a Red^+^ cell (Fig. 3F, green arrow). Thus, while the initial staining process resulted in intracellular stain, likely due to membrane invagination, cells which acquired the faint staining as a result of PtxM treatment only visibly displayed the second membrane stain associated with the cell-surface. However, we cannot rule out the possibility that transfer of membrane units too small to be visualized by light microscopy makes a significant contribution to the overall staining intensity observed by flow cytometry.

### Cellular Processes Involved in Aggregation and Membrane Transfer

**Figure 4:**
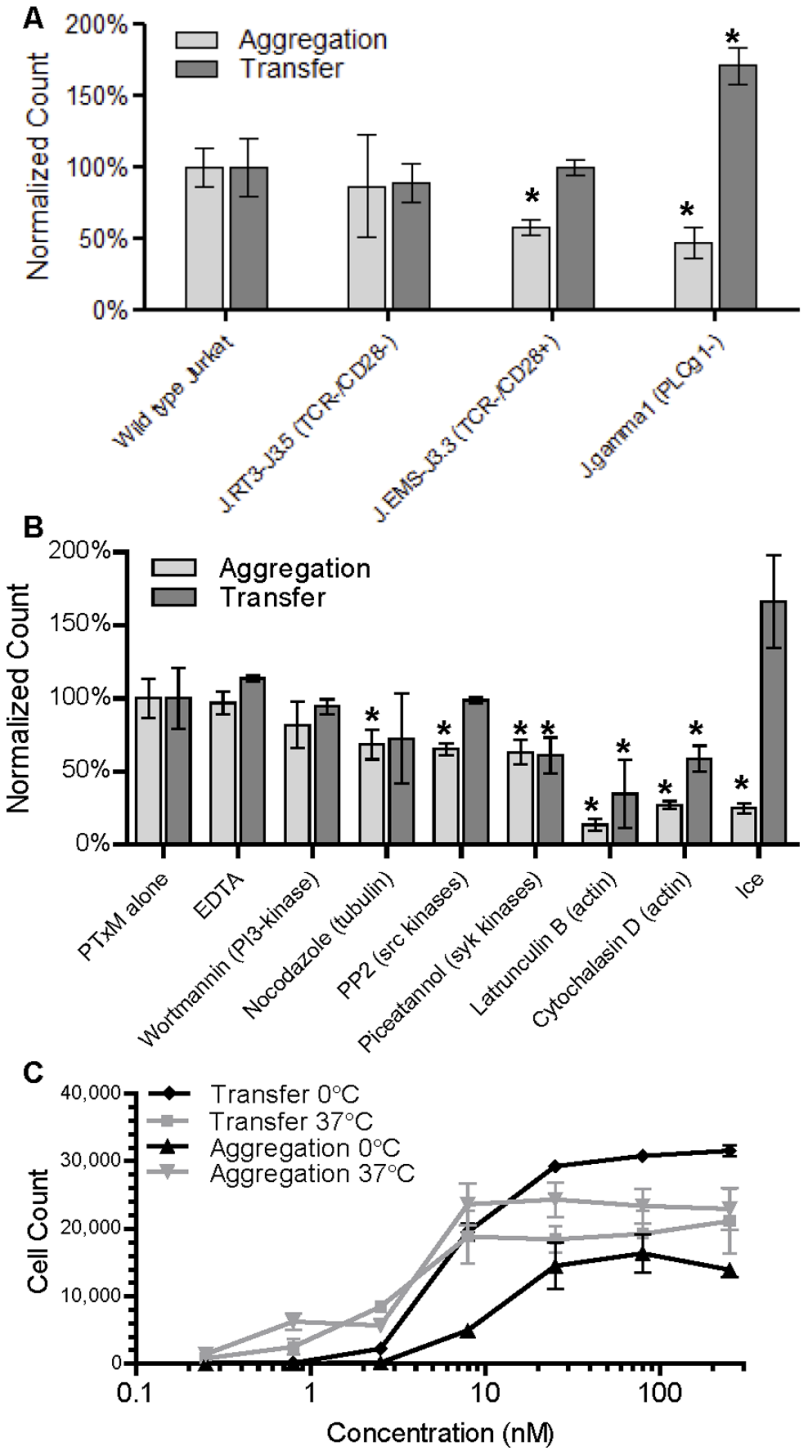
Effect of inhibitors and mutation on aggregation and transfer. **A.** Comparison of PTxM treatment (7.9 nM for 1 hr at 37°C) of Jurkat cell lines and derivatives, E6-1 (wild type), J.RT3-J3.5 (TCR−/CD28-), J.EMS-J3.3 (TCR−/CD28+), and J.gamma1 (PLC-γ1-), for aggregation (light grey) and transfer (dark grey). Population sizes are normalized to the mean wild type aggregation or transfer. Results represent the mean of three independent assays with standard deviation. * indicates significant difference from wild type by Student’s T-test (P<0.05). **B.** Comparison of PTxM treatment (7.9 nM for 1 hr at 37°C) of Jurkat cells pretreated under differing conditions for aggregation (light grey) and transfer (dark grey). Population sizes are normalized to the mean aggregation or transfer under non-pretreated conditions (PTxM alone). Results represent the mean of three independent assays with standard deviation. * indicates significant difference from PTxM alone by Student’s T-test (P<0.05). **C.** A dose- response study examining the amount of aggregation versus membrane transfer as a function of PTxM concentration at 37°C and on ice. Results represent the mean of three independent assays with standard deviation.

Signaling through the TCR is known to be activated by PTx independently of the enzymatic activity of the A subunit [3]. To determine whether the membrane transfer phenomenon requires this signaling activity, we evaluated the ability of PTxM to mediate membrane transfer in several TCR pathway mutants (Fig. 4A). Interestingly, the Jurkat mutant J.RT3-J3.5, which lacks both TCR and CD28 expression [11], [12], did not exhibit any differences in aggregation or membrane transfer as compared to the parental, wild type Jurkat cells, suggesting that neither cell-surface signaling molecule is required. However, the Jurkat mutant J.EMS-J3.3, which lacks TCR expression but does express CD28 [11], [13], displayed reduced aggregation. The reason for the differences in PTxM-mediated aggregation between J.RT3-J3.5 cells and J.EMS-J3.3 is unclear, but may result from non-TcR-related differences between these mutagenized cell lines. The Jurkat mutant J.gamma1, which lacks PLCγ1 expression due to a frame-shift mutation, demonstrated significantly reduced aggregation while membrane transfer was significantly enhanced.

The phenotype of the J.gamma1 mutant led us to examine the possible involvement of other signaling pathways, by evaluating cells pretreated with pharmacologic agents (Fig. 4B). The inhibitors EDTA (a metal ion chelator which disrupts calcium-dependent signaling) and wortmannin (a PI 3-kinase inhibitor) did not significantly alter either aggregation or membrane transfer. Nocodazole (which promotes tubulin depolymerization), PP2 (a src kinase inhibitor), and piceatannol (a syk kinase inhibitor) caused only modest decreases in either aggregation or membrane transfer, suggesting that these signaling systems are not essential for aggregation or transfer. In contrast, two actin polymerization inhibitors, latrunculin B and cytochalasin D, significantly reduced both aggregation and membrane transfer. Aggregation was more severely affected than membrane transfer, and was nearly eliminated in the presence of latrunculin B. None of the inhibitors alone (i.e., in the absence of PTx) promoted either aggregation or membrane transfer. Since the confocal studies demonstrate that aggregated cells display close contact over a very large cell-surface area, association likely involves membrane rearrangements mediated by actin remodeling. Agents that affect actin polymerization would inhibit the ability to generate such close cellular contact. Consistent with this, when PTxM was added to cells on ice, aggregation was significantly reduced (Fig. 4B). The ability of incubation on ice to inhibit all cellular processes requiring ATP, including actin-remodeling, could account for the reduced cellular aggregation. Interestingly, however, membrane transfer appeared to be enhanced when cells were incubated on ice. The ability of both low temperature and deletion of PLCγ to reduce aggregation, while simultaneously enhancing membrane transfer, further supports the hypothesis that aggregation is not an obligatory precursor to membrane transfer, as suggested by the time course studies demonstrating that membrane transfer and cellular aggregation follow a similar time course (Fig. 2F). Moreover, these data indicate that whereas aggregation is an active process involving signaling and metabolic activity, membrane transfer is largely passive.

The increased membrane transfer for cells incubated on ice is difficult to reconcile with the reduced membrane transfer seen with the actin inhibitors. A possible explanation is that membrane transfer may occur by more than one mechanism, which may have different requirements for optimum efficiency, an explanation also supported by the confocal studies. Some confocal images visualized very large vesicles (>2 µm) attached to the cell membrane. However, some images revealed small areas of contrasting stain (<0.5 µm). Large particles may require actin remodeling for tight association, whereas small membrane vesicles may be transferred without actin remodeling. Nevertheless, both aggregation and membrane transfer showed a similar dependence on PTx concentration, both at 37° and on ice (Fig. 4C).

### Direct Cell to Cell Contact is not Required for Membrane Transfer

**Figure 5:**
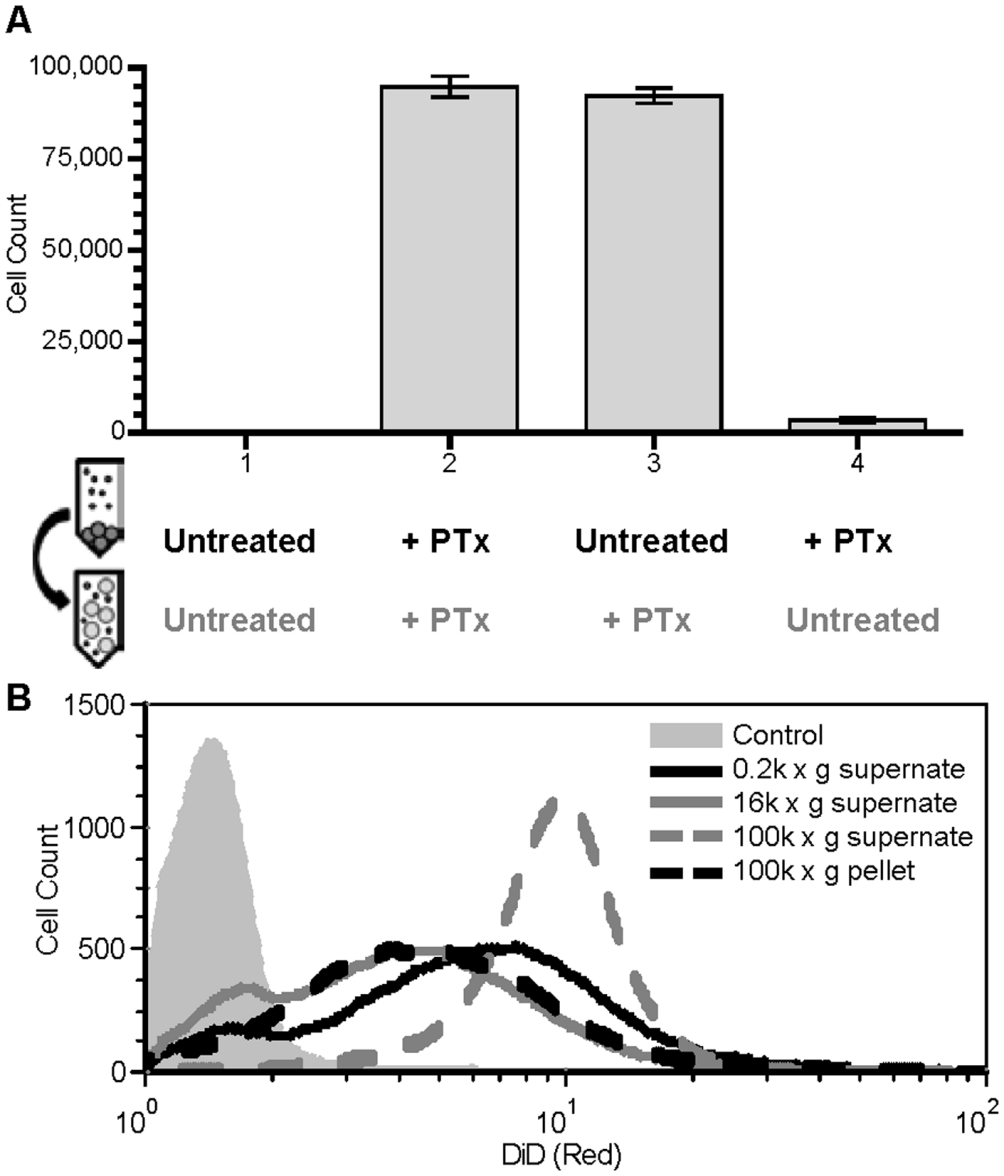
Cell-to-cell contact is not required for membrane transfer. **A.** Effect on transfer of cell depleted supernatant, from PTxM-treated or untreated Green^−/^Red^+^ Jurkat cells, added to a population of PTxM treated or untreated Green^+^/Red^−^ Jurkat cells with schematic representations of protocols below related data. Results represent the mean transfer population size of three independent assays with standard deviation. **B.** Effect on transfer of differentially centrifuged cell depleted supernatant from untreated Green−/Red+ Jurkat cells, added to a population of PTxM treated Green+/Red- Jurkat cells. Untreated Green+/Red- Jurkat cells with 200×g supernatant (grey field) and PTxM treated Green+/Red- Jurkat cells with 200×g supernatant (black solid line), 16,000×g supernatant (dark grey solid line), 100,000×g supernatant (dark grey dashed line), or the resuspended pellet from the100,000×g supernatant (black dashed line).

We examined the process of membrane transfer in further detail to determine whether PTx is required to mediate liberation of membrane fragments from the donor cell, or for the capture of these fragments by the recipient cell (or both). Red- and Green-stained cells were separately incubated with or without PTxM on ice, conditions which promote membrane transfer and inhibit aggregation. Whole cells were removed from the Red-stained population by centrifugation at 200×g for 10 minutes, and the Red cell-depleted supernatant was then added to intact Green cells and analyzed by flow cytometry for the presence of Red membrane transfer to the Green cells (Fig. 5).

No membrane transfer was seen when both the Red and Green cells were left untreated (Fig. 5A, condition 1). In contrast, significant membrane transfer was seen when both the Red-stained supernatant and the intact Green cells were treated with PTxM (Fig. 5A, condition 2). These results demonstrate that membrane transfer can occur in the absence of direct cell to cell contact (i.e., in the absence of intact donor cells).

When only the intact Green (recipient) cells were treated with PTxM, membrane transfer was as efficient as when both populations were treated (Fig. 5A, condition 3). In contrast, when only the Red (donor) cells were treated with PTxM, membrane transfer was essentially absent (Fig. 5A, condition 4). The ability to detect efficient membrane transfer using supernatant isolated from cells not treated with PTxM demonstrates that membrane particles are generated independently of PTxM-treatment and that PTxM treatment is not required for liberation of such particles; however, PTxM is required for the transfer of these membranes to intact cells.

We used differential centrifugation to determine the approximate size of vesicles required for mediating membrane transfer. After the initial slow speed centrifugation (500×g), untreated red-stained cells were centrifuged for a second time at 16,000×g, and the resulting supernatant was divided and one aliquot was re-centrifuged at 100,000×g prior to addition to PTxM-treated acceptor cells. Membrane transfer was observed with the supernatant from both the 16,000×g spin and the 100,000×g spin, as well as with the pellet from 100,000×g spin (Fig. 5B), suggesting that vesicles of varying sizes can be transferred. Indeed, confocal microscopy of cells incubated with the 16,000×g supernatant revealed small punctate red staining associated with the surface of Green+ cells, but lacked the larger extracellular spheres seen in Fig. 3 (data not shown). Moreover, the intensity of red staining in the Green+ populations treated with the low-speed or medium-speed supernatants, or with the high-speed pellet, is quite heterogeneous, again presumably reflecting the heterogeneity in the sizes of the vesicles being transferred (Fig. 5B). Interestingly, however, the intensity of red staining is more homogeneously high when the 100,000×g supernatant is used. Since the vesicles in this fraction are too small to be visualized by light (confocal) microscopy, these results suggest that many more vesicles of this size are transferred per recipient cell in order to achieve this intensity of staining. This in turn implies that such vesicles are either much more abundant in the original supernatant, or are transferred to the recipient cell much more efficiently, or both. In sum, these results suggest that membrane transfer can occur with a wide diversity of sizes of membrane particles, including and especially those too small to be visualized by light microscopy.

### Comparison of PTxM to Other Mitogenic Lectins

Lectins, including Ptx, recognize the sugars that decorate glycoproteins and glycolipids expressed on the mammalian cell-surface. The N-linked glycans are typically complex mixtures of sugars attached to a branched mannose-containing core. O-linked glycans contain only a few sugars and lack mannose. Glycolipids typically contain sialic acid, N-acetylgalactosamine, D-glucose or D-galactose.

**Figure 6:**
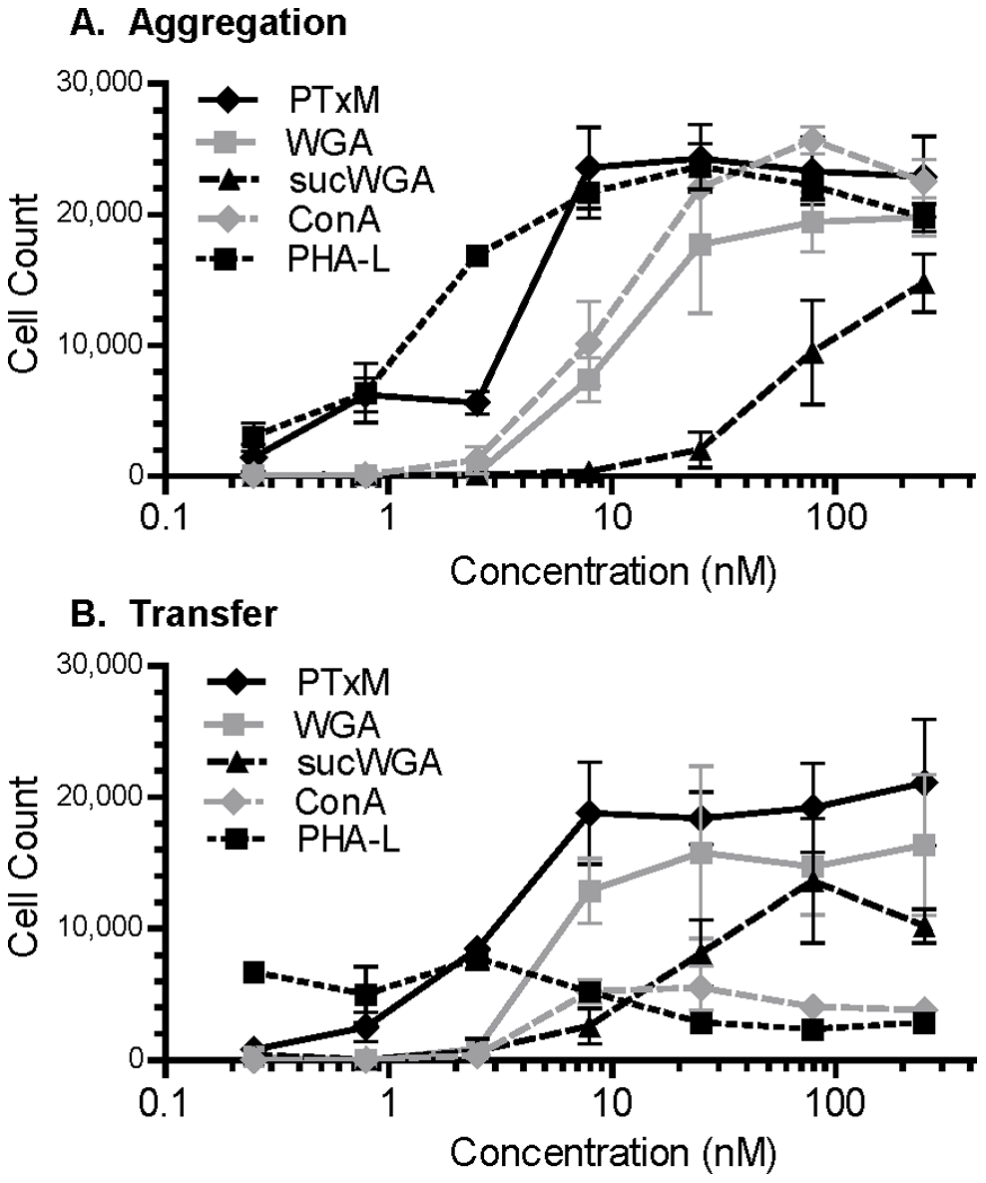
Effect of differing concentrations of lectin on Jurkat cell aggregation and membrane transfer population sizes. Results represent the mean of three independent assays with standard deviation. **A.** Jurkat cell aggregation. **B.** Jurkat cell membrane transfer.

We investigated several plant lectins with known T cell activity for their ability to mediate aggregation and membrane transfer. PTxM-mediated aggregation occurred at an effective concentration for 50% (EC_50_) of 3.2 nM (Table 1). Each of the plant lectins tested also induced aggregation, with the rank order (best to worst) PHA-L, ConA, WGA, and sucWGA (Fig. 6A, Table 1). In contrast, when membrane transfer was examined, only PTxM, WGA, and to a lesser extent sucWGA were able to mediate membrane transfer events (Fig. 6B). Interestingly, the plant lectins that were most efficient at mediating aggregation, PHA-L (EC_50_, 1.3 nM) and ConA (EC_50_, 11 nM), were unable to mediate membrane transfer. In contrast, sucWGA, which was very inefficient at mediating aggregation (EC_50_, 154 nM), was able to mediate membrane transfer at much lower concentrations (EC_50_, 18 nM).

**Table 1:** Activity of PTx and Lectins on Aggregation and Membrane Transfer in Jurkat cells.

Lectin	Preferred Ligands^a^	TCR activation^b^	Aggregation EC_50_ (nM)	Transfer EC_50_ (nM)
PTx	NeuAc, Complex N-glycans	N and O	3.2	3.1
WGA	GlcNAc, NeuAc	N and O	14	6.4
sucWGA	GlcNAc	N and O	154	18
PHA-L	Galβ1-4GlcNAcβ1-2Man	N	1.3	>250
ConA	Branched (Manα)_n_	N	11	>250

a NeuAc (Sialic acid), GlcNAc (N-acetylglucosamine), Gal (galactose), Man (mannose).

b Glycan type (N-linked or O-linked) recognized by the lectin when promoting activation of the T cell receptor.

In a recent study [4], the ability of PTx and plant lectins to activate the T cell signaling pathway through native CD3 containing N-linked glycans, or a receptor engineered to express only O-linked glycans was evaluated. As summarized in Table 1, the lectins that use N-linked glycan binding to activate the TCR pathway (ConA and PHA-L) were unable to promote membrane transfer. In contrast, the three lectins (PTxM, WGA, and sucWGA) that can activate the TCR signaling pathway through either N-linked or O-linked glycan binding were all capable of mediating membrane transfer. Both PTxM and WGA can bind sialic acid while sucWGA cannot, suggesting that the capacity to bind sialic acid may not be required for membrane transfer [14], [15]. These results strongly support the hypothesis that membrane transfer is not mediated through recognition of N-linked glycans. However, it does not mean that membrane transfer is mediated through O-linked glycans, since the same sugars displayed on O-linked glycans are also displayed on glycolipids. Glycolipids are frequently found in cholesterol-rich lipid raft microdomains, specialized membrane compartments that play an important role in cell-signaling and protein trafficking. Since transfer involves membrane particles, ability of the transfer-proficient lectins (WGA, sucWGA and PTx) to bind to glycolipids provides an attractive explanation for this process.

### Mapping the Binding Sites on PTx Required for Activity

To determine which subunits and binding sites on the PTxB pentamer are necessary for PTx-mediated aggregation and membrane transfer, we assessed the activity of a panel of recombinant PTx B subunits [16], some of which have mutations in known glycan recognition sites. The glycan recognition regions on PTx have been localized to the S2 and S3 subunits, with each possessing two binding regions. The C-terminal binding sites of S2 and S3 are well characterized and recognize sialic acid. The N-terminal binding sites are less well-defined, and likely recognize short chain oligosaccharides. The S4 subunit, which lacks glycan binding sites, is stable and the monomeric form was purified. However, the binding subunits S2 and S3 are not stable in the absence of S4, so wild type S2S4 and S3S4 were purified as heterodimers. Dimers with mutations in the C-terminal sialic acid (SA) binding site are designated ΔSA-S2S4 and ΔSA-S3S4.

**Figure 7:**
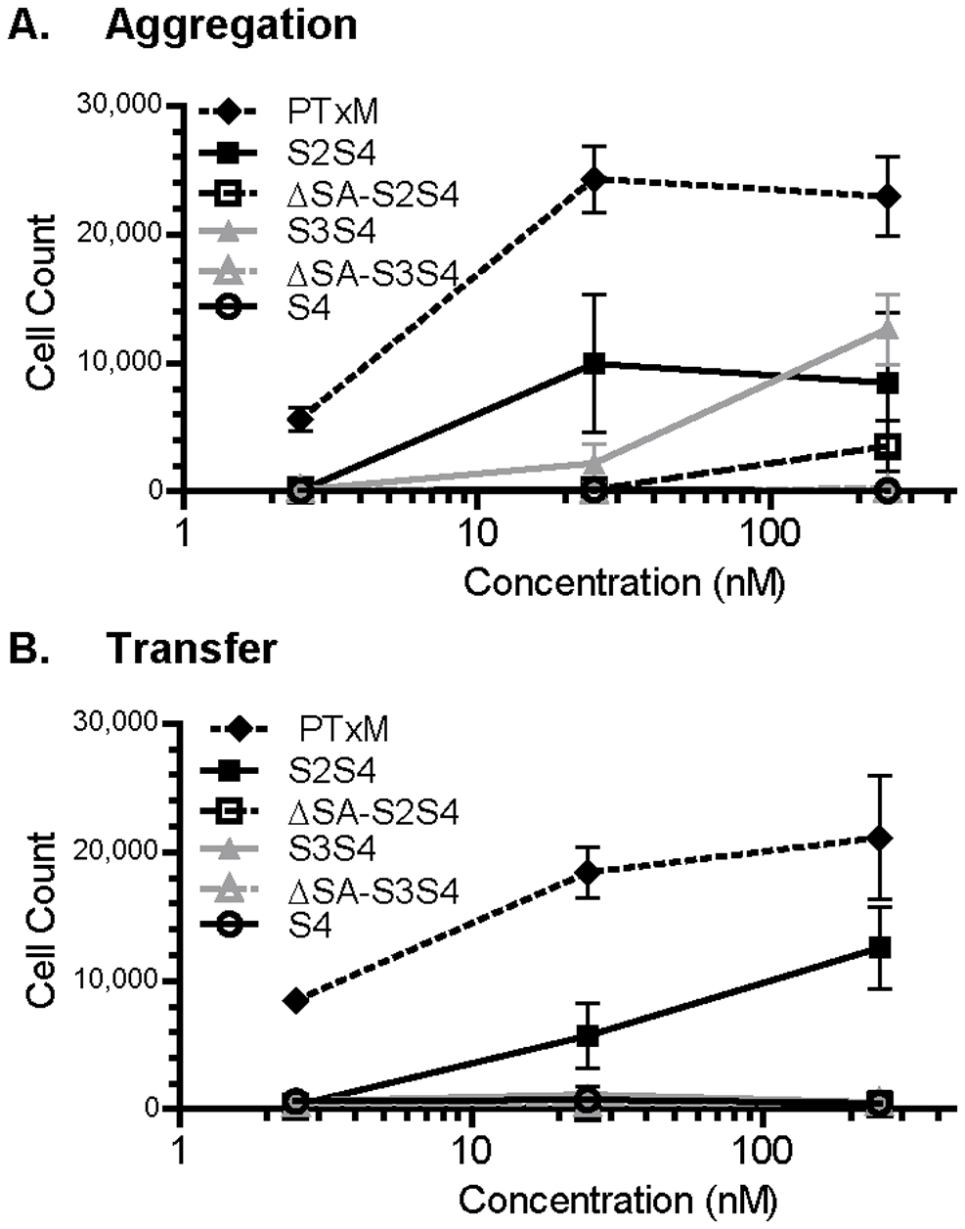
Effect of differing concentrations of recombinant PTxB subunit dimers on Jurkat aggregation and membrane transfer. Results represent the mean of three independent assays with standard deviation. **A.** Aggregation. **B.** Membrane transfer.

Both the S2S4 and S3S4 dimers mediated aggregation (Fig. 7A), although intact PtxM (with four glycan binding regions) was able to promote a higher level of aggregation than the dimers. However, aggregation mediated by PTxM and the S2S4 dimer occurred at similar concentrations, while aggregation mediated by the S3S4 dimer required higher concentrations. These results suggest that aggregation of Jurkat cells is largely mediated by the binding sites on the S2S4 dimer. The S4 monomer control did not demonstrate any ability to cause aggregation (Fig. 7), consistent with the glycan array studies demonstrating that S4 lacks glycan binding sites [16]. The ΔSA-S2S4 and the ΔSA-S3S4 dimers also failed to promote aggregation, consistent with the idea that lectins require two binding sites in order to crosslink two separate cells. The ability of ΔSA-S2S4 to mediate aggregation at high concentrations could be due the complexity of the N-terminal binding site and its ability to engage oligosaccharides (as opposed to single sugars)- it is formally possible that this single site could simultaneously, if inefficiently, engage and crosslink sugars on different molecules.

When membrane transfer was examined (Fig. 7B), only PTx and the S2S4 dimer were observed to have activity, although the activity for the S2S4 dimer was considerably reduced compared to PtxM. These results suggest that the S2S4 dimer is also primarily responsible for mediating membrane transfer. Both WGA and S2 support membrane transfer, while S3 does not, and it is interesting to note that S2 (but not S3) contains a domain with homology to the lectin WGA [17]. This binding region likely plays an important role in mediating membrane transfer.

**Figure 8:**
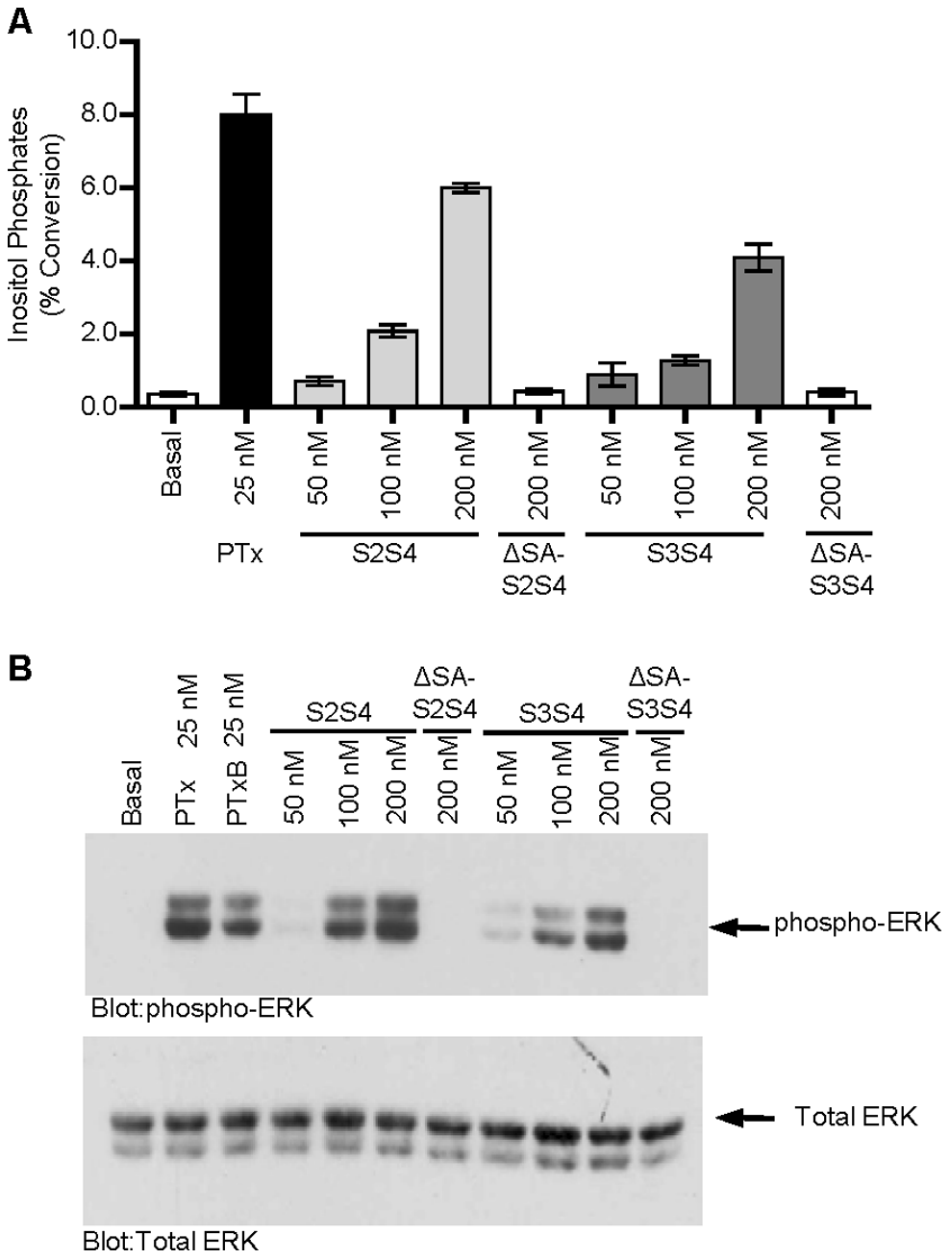
Recombinant S2S4 and S3S4 PTx Dimers are Robust Activators of Signal Transduction in Jurkat cells. Recombinant S2S4 and S3S4 PTx dimers were purified from *E. coli* and tested for their abilities to activate PLC and the MAP kinase ERK. S2S4 and S3S4 dimers containing mutations in the conserved carbohydrate binding domain (ΔSA-S2S4 and ΔSA-S3S4) were also similarly tested. **A.** Wild type Jurkat cells were stimulated with the indicated concentrations of dimers and PLC activity was measured using the inositol phosphate accumulation assay. **B.** Wildtype Jurkat cells were stimulated with the indicated concentrations of dimers and ERK activity was measured by western blot using phospho-specific ERK antibodies.

PTxM is known to activate the TCR signaling pathway [3]. To ensure that the lack of transfer activity seen with the S3S4 dimer is not due to defective assembly or conformation, we assessed the ability to of the dimers to activate phospholipase C gamma (PLC-γ) and the MAP kinase, ERK. Wild type Jurkat cells were stimulated with the indicated concentrations of PTx B subunit dimers, and PLC activity was measured using the inositol phosphate accumulation assay (Fig. 8A). While not as potent as PTx holotoxin, both S2S4 and S3S4 dimers promoted a dose-dependent increase in inositol phosphate accumulation, while the ΔSA-S2S4 and ΔSA-S3S4 mutant forms lacked activity. Similarly, both S2S4 and S3S4 dimers promoted a dose-dependent increase in phospho-specific ERK, and again, the ΔSA-S2S4 and ΔSA-S3S4 mutant forms lacked activity. These results indicate that carbohydrate binding sites present in either the S2/S4 or S3/S4 dimer are sufficient to promote signaling in T-cells. While the S3S4 dimer may have slightly less activity than the S2S4 dimer in the T cell activation assays, these studies clearly rule out the possibility of any gross structural defect of the S3S4 dimer being responsible for its observed inability to mediate membrane transfer. These results are also consistent with the observation that the lectins ConA and PHA-L can also activate the TCR, but do not promote membrane transfer.

### PTxM Mediated Membrane Transfer in Other Cell Lines

Since lectins, including PTx, mediate aggregation in diverse cell types, we wanted to determine if membrane transfer also occurs in other cell types. PTx is known to activate signaling cascades in Chinese hamster ovary (CHO) cells, leading to a clustering response due to a failure of daughter cells to separate after division [18]. However, the cell clustering phenotype requires the enzymatic activity of S1, which is absent in PTxM [19].

**Figure 9:**
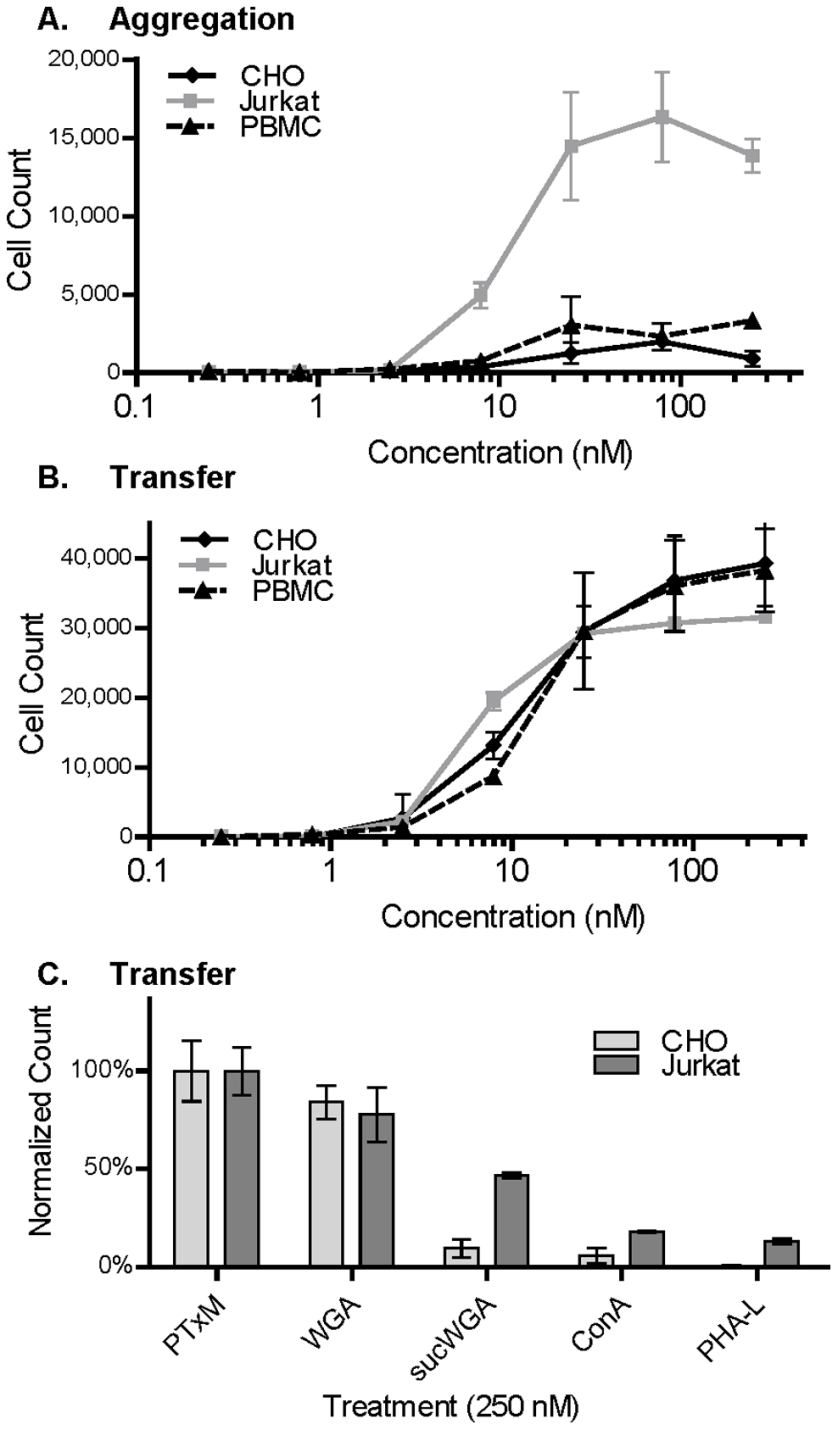
Effects of PTxM or lectin treatment on CHO cells, Jurkat cells, and PBMCs. Cells were treated with the indicated concentrations of PTxM or lectins for 1 hr on ice, and the number of cell aggregates (**A**) and cells demonstrating membrane transfer (**B, C**), were determined by flow cytometry. Results represent the mean of three independent assays with standard deviation. In **C**, membrane transfer population sizes for lectin treatment were normalized to the mean PTxM transfer population size for each cell type.

PTxM-mediated aggregation and membrane transfer in CHO cells was compared to that in Jurkat T cells (Fig. 9). Unlike Jurkat cells, CHO cells are adherent, so trypsin was used to detach the cells, and aggregation and transfer studies were performed on ice to prevent reattachment. PTxM mediated very little aggregation of CHO cells under these conditions (Fig. 9A), although aggregation was seen in Jurkat cells under the same conditions. In contrast, robust membrane transfer was observed for both cell types (Fig. 9B), with similar dose-response characteristics. The ability of plant lectins to promote membrane transfer in CHO cells was also examined (Fig. 9C). For both Jurkat and CHO cells, WGA mediated efficient membrane transfer, while sucWGA, ConA and PHA-L were much less efficient at promoting membrane transfer (although in these latter cases, membrane transfer was slightly higher for Jurkats as compared to CHO cells). Studies similar to those described in Fig. 5 were also performed using CHO cells; cell to cell contact was not needed for PTxM-mediated membrane transfer in CHO cells, and membrane transfer occurred from CHO cell donors to Jurkat cell acceptors, and vice versa (data not shown). Membrane transfer also occurred in the Vero African green monkey kidney cell line (data not shown).

### Membrane Transfer Includes Membrane Proteins in Primary Human Cells

**Figure 10:**
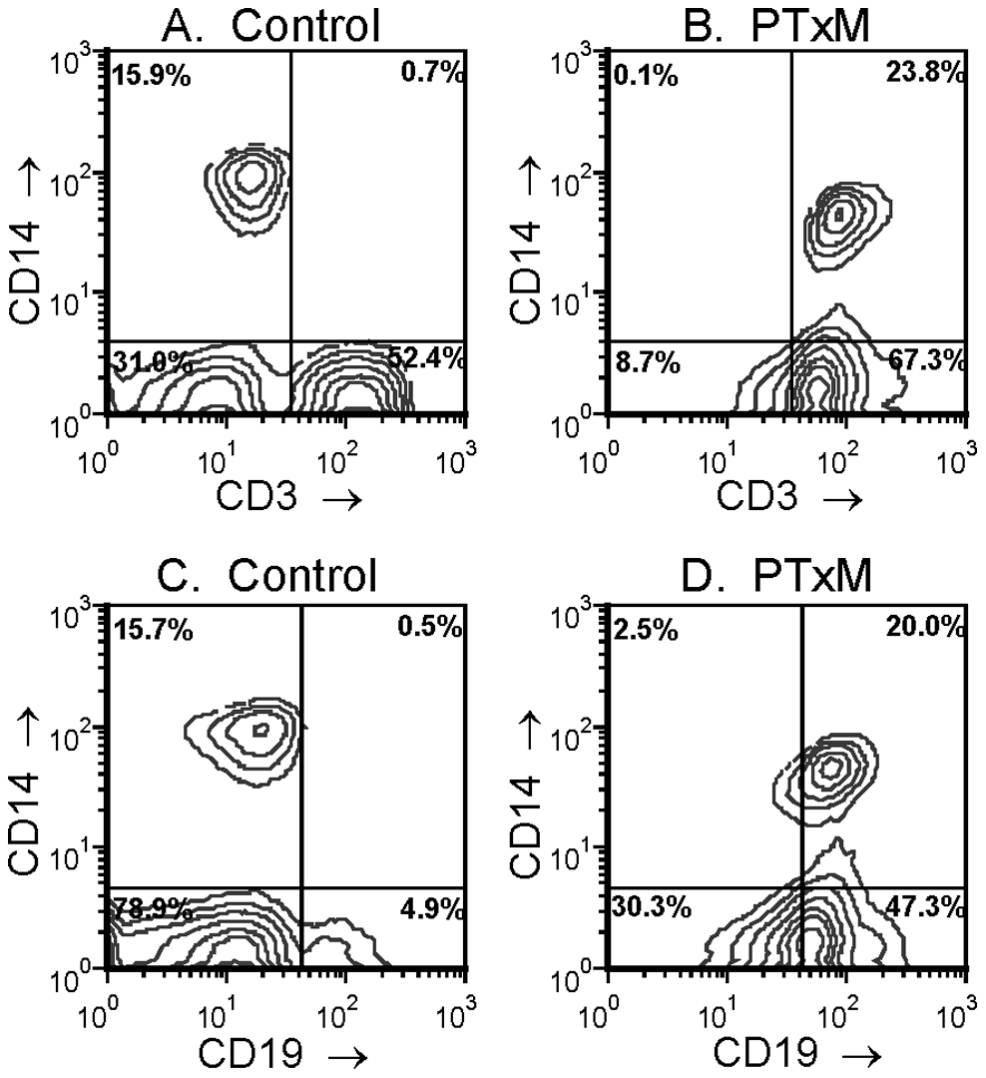
PTxM-mediated intercellular transfer of membrane proteins. Peripheral blood mononuclear cells (PBMC) were isolated from human donors, and stained for CD3 (a T cell marker), CD19 (a B cell marker), and CD14 (a monocyte cell marker). **A.** Untreated cells showing CD3 vs CD14. **B.** PTxM pretreated cells showing CD3 vs CD14. **C.** Untreated cells showing CD19 vs CD14. **D.** PTxM pretreated cells showing CD19 vs CD14.

Since all of the studies to this point were performed with immortalized cell lines, we wanted to determine if PTxM could mediate membrane transfer in primary human cells and whether, in addition to membrane lipids, integral membrane protein markers were also transferred from one cell type to another. Peripheral blood mononuclear cells (PBMC) were isolated from human donors, and stained for CD3 (a T cell marker), CD19 (a B cell marker), and CD14 (a monocyte cell marker). These studies were performed on ice to prevent attachment of monocytes (Fig. 10), as well as at 37°C (data not shown). PBMCs displayed reduced aggregation compared to Jurkat cells (Fig. 9A), similar to CHO cells. Membrane transfer was observed with roughly equal efficiency in PBMCs as compared to Jurkat and CHO cells (Fig. 9B). Staining of the untreated population of PBMC with anti-CD3 and anti-CD14 demonstrates the presence of three major cell populations: CD3^+^/CD14^−^ T cells (Fig. 10A lower right quadrant), CD3−/CD14+ monocytes (upper left quadrant), and CD3^−/^CD14^−^ cells (lower left quadrant). When these cells were pre-treated with PTxM, changes in the staining pattern were evident. An increase in CD3 staining (shift to the right) was seen for both the CD3^−/^CD14^−^ cells (which now overlap with the T cell population, lower cluster in Fig. 10B), and the CD3^−/^CD14^+^ monocytes (upper cluster in Fig. 10B). The CD3^+^ T cell population stained less brightly with anti-CD3 after PTxM treatment; however, we have previously shown that PTx binds to and activates T cells via their T cell receptor molecules [4]. Thus, it is likely that the reduced staining with anti-CD3 reflects toxin-mediated aggregation of CD3, simple steric inhibition of antibody binding by the toxin, or both. Similarly, reduced staining for CD14 was observed PTxM-treated monocytes, and PTx has also been shown to bind to CD14 [20]. We conclude from these experiments that CD3 has been transferred to both populations of cells that were originally CD3-negative in the starting population. There is also evidence for the acquisition of CD14 by the CD14-negative cells (shift upward in Fig. 10B as compared to Fig. 10A). However, this shift is not nearly as pronounced as the shift for CD3.

A very similar pattern is seen with CD19 staining for B cells. Staining of the untreated population of PBMC demonstrates the presence of three major cell populations: CD19^+^/CD14^−^ B cells (Fig. 10C lower right quadrant), CD19^−/^CD14^+^ monocytes (upper left quadrant), and CD19^−/^CD14^−^ cells (lower left quadrant). When these cells were pre-treated with PTxM, an increase in CD19 staining is seen for both of the other (originally CD19^−^) populations (Fig. 10D). It should be noted here that the apparent overall increase in CD19 staining presumably results from the transfer of CD19 from small membrane vesicles (which are too small to be detected as cells by the instrument, and hence do not show up on the plots) to intact cells as a result of PTx treatment.

Transfer of CD14 to the lymphocyte populations is less robust than CD3 or CD19 transfer, possibly because CD14^+^ cells do not efficiently produce the appropriate membrane vesicles, or such vesicles are produced but fail to be targeted to lymphocytes. Alternatively, appropriate vesicles may be produced and bind to lymphocytes, but CD14 is excluded from them. While we cannot at present distinguish between these possibilities, these results do clearly demonstrate that membrane protein transfer between cell populations can occur using freshly isolated normal human hematopoetic cells. It should be noted that the conditions used for the assay shown in Fig. 10 (1 hr treatment on ice) rule out the possibility that PTxM is inducing aberrant expression of these markers via signal transduction and new transcription/translation.

In conclusion, we have demonstrated that not only does PTx cause aggregation in many cell types, including the Jurkat T cell lymphoma line, it also mediates the intercellular transfer of both membrane and protein material. PTx mediated membrane transfer does not require direct cell to cell contact, and treatment of only the recipient cell with PTx is sufficient for membrane transfer. PTxM is not essential for release of the relevant membrane vesicles, although we cannot rule out the possibility that PTxM may enhance some types of membrane release.

Membrane transfer by PTx appears to be a passive, lectin-like cross-linking event between membranes released from donor cells and an acceptor cell, and can be replicated with the plant lectin, WGA. The S2 subunit of PTx, which shares homology with WGA, seems to be largely responsible for both aggregation and transfer. Curiously, while capable of inducing aggregation, neither ConA nor PHA-L could mediate transfer. Membrane transfer appears to require the ability to bind to O-linked glycans or glycolipids, or both, a property shared by PTx, WGA and S2S4 dimer.

The composition, nature, and origin of the transferred membrane units remains unclear. The membrane particles attached to cells via PTxM are very heterogeneous in size and likely in protein composition, and are probably generated by diverse cellular processes. A variety of membrane vesicles arising from eukaryotic cells have been described, which differ in their lipid and protein components, and which appear to be derived from different cellular membranes. Dying and apoptotic cells release larger membrane fragments (up to 500 nm diameter) that can be sedimented at 10,000×g [21]. Such vesicles are not entirely responsible for the transfer phenomena demonstrated here, since centrifugation even at 100,000×g failed to deplete cell supernatants of activity. Smaller vesicles include exosomes (50–100 nm) or exosome-like vesicles (20–50 nm) [21]. Exosomes, which are secreted by fusion of multivesicular endosomes with the plasma membrane, are constitutively secreted by many cell types, including most tumor cells lines as well as dendritic cells, macrophages, and activated T and B cells. Exosomes can contain antigens and antigen-MHC complexes that are capable of inducing antigen-specific T cell responses *in vitro* [21], or, under certain conditions, may inhibit antigen (tumor)-specific responses [22]. Interestingly, recent work [23] demonstrated that a cytosolic/nuclear protein phosphatase and tumor suppressor, PTEN (phosphatase and tensin homologue deleted on chromosome 10), is specifically targeted into and secreted via endosomal vesicles. These endosomes were shown to be capable of delivering functionally active (cytosolic) PTEN to intact target cells. Moerover, exosomes have recently been shown to contribute to the function of regulatory T cells [24].

Aberrant targeting of such vesicles may be relevant to the pathogenic activity of *Bordetella pertussis*. We envisage two non-mutually exclusive mechanisms by which this may occur. First depending on the cell type, mis-targeted vesicles may induce the activation of antigen non-specific immune cells, reminiscent of superantigens, or may directly induce antigen non-responsiveness in antigen-specific T cells, similar to what has been documented for tumor-specific responses [22]. Secondly, and perhaps more likely, due to the promiscuous nature of PTx binding activity, PTx may aberrantly target functional vesicles to irrelevant cellular targets, thereby preventing them from reaching their intended target cells. Thus, PTx may act *in vivo* as a negative regulator of exosome function by depriving the normal physiological target of exosomal content that is required for normal physiological function.

Importantly, our observations demonstrate vesicle-mediated transfer of membrane and protein components under the conditions used does not spontaneously occur in the absence of PTx, and that protein markers embedded in membranes of primary human PBMC can also be transferred in a PTxB-dependent manner. Aberrant targeting of such vesicles may be relevant to the pathogenic activity of *Bordetella pertussis*. In addition, while the enzymatic activity of PTx has been eliminated in the currently used acellular pertussis vaccine formulations, it is not clear whether the lectin activity of the B-pentamer has also been eliminated, and whether residual activity could play a role in reducing vaccine efficacy. PT concentrations range from 2.5 to 25 µg per half mL dose in these vaccines (corresponding to 50–500 nM), which far exceeds the dose needed to promote membrane exchange.

## Materials and Methods

### Cells and Reagents

Jurkat cell lines and derivatives, E6-1 wild type [25], J.gamma1 (PLC-γ1^−^) [26], J.EMS-J3.3 (TCR^−/^CD28^+^) [12], [25], and J.RT3-J3.5 (TCR^−/^CD28^−^) [13], [25], were maintained in RPMI (Gibco/Invitrogen, Carlsbad, CA) containing 10% fetal bovine serum (Gibco), 50 units mL^−1^ of penicillin, and 50 µg mL^−1^ of streptomycin (Gibco) at a cell density between 2×10^5^ and 1×10^6^ cells mL^−1^. Chinese hamster ovary (CHO) cell line K-1 [27] was maintained in F-12 containing 10% fetal bovine serum (Gibco) and 1% penicillin-streptomycin (Gibco).

Human Peripheral Blood Mononuclear Cells (PBMC) were isolated from whole blood centrifuged over Ficoll-Paque as previously described [2]. Cells were adjusted to 10^6^ cells mL^−1^ in PBS pH 7.4, equilibrated to 4° or 37°C, and incubated in the presence or absence of PTxM (250 nM) at the same temperature. Cells were stained with FITC-labeled αCD3 and APC labeled αCD14 (Miltenyi Biotec Inc., Cologne, Germany) and incubated 1 hr at 4°C. Relative staining with the two markers was assessed by flow cytometry.

Genetically toxoided PTx (PTxM) carrying the S1 9K/129G double mutation was prepared at Chiron Bioscience and kindly provided by Rino Rappuoli [28]–[30]. The recombinant S2S4 dimer, S3S4 dimer, mutant ΔSA-S2S4 dimer, mutant ΔSA-S3S4 dimer, and S4 monomer were produced as previously described [16]. The lipophilic florescent dyes, DiO (3,3'-dioctadecyloxacarbocyanine perchlorate) and DiD (1,1'-dioctadecyl-3,3,3',3'-tetramethylindodicarbocyanine perchlorate), and the intracellular amine-coupled fluorophore CFDA-SE (carboxyfluorescein diacetate succinimidyl ester) were purchased from Molecular Probes/Invitrogen (Carlsbad, CA). Latrunculin B (from *Latrunculia magnifica*), cytochalasin D (from *Zygosporium mansonii*), PP2 (4-Amino-5-(4-chlorophenyl)-7-(*t*-butyl)pyrazolo[3,4-d]pyrimidine), nocodazole (Methyl-[5-(2-thienylcarbonyl)-1H-benzimidazol-2-yl]-carbamate), piceatannol (*trans*-3,3′,4,5′-Tetrahydroxystilbene), and wortmannin (11-(acetyloxy)-1S,6bR,7,8,9aS,10,11R,11bR-octahydro-1- (methoxymethyl)-9a,11b-dimethyl-3H-furo[4,3,2-de]indeno[4,5- h]-2-benzopyran-3,6,9-trione) were purchased from Calbiochem/EMD (Gibbstown, NJ). Wheat-germ agglutinin (WGA) from *Triticum vulgaris*, succinylated wheat germ agglutinin (sucWGA), concanavalin A (ConA) from *Canavalia ensiformis*, and Phytohemagglutinin leucoagglutinin homotetramer (PHA-L) from *Phaseolus vulgaris* were purchased from Vector Laboratories (Burlingame, CA).

### Cell Staining

For staining with the lipophilic dyes DiO and DiD, cells were harvested into complete culture medium and adjusted to 1×10^6^ cells mL^−1^. Cells were stained with DiO at 5 µM or 10 µM as indicated, or DiD at 5 µM. A 1 mM solution of a dye was added to a 15 mL conical tube at a dosage of 5 µL per mL of cells, yielding a final dye concentration of 5 µM (unless noted otherwise in text). The cell suspension was added rapidly to the tube with dye and mixed by inversion several times. The cells were then incubated at 37°C for 15 minutes on a rocker and protected from light. Cells were then pelleted by centrifugation at 200×g for 5 minutes, washed twice in serum-free medium, and adjusted to 1×10^6^ cells mL^−1^ with same. Equal volumes of DiO and DiD stained cells were combined, mixed, and stored at 37°C or on ice, depending on experimental design. The total time between the first serum-free media wash and the addition of PTxM (or lectins) for flow cytometry experiments was 30 minutes.

### Ethics Statement

Primary human cells were collected with written consent from the donor and with approval from the Institutional Review Board of the University of Cincinnati (Protocol #93-05-20-01-E, “Human Serum and Protection against *Bordetella pertussis*).

### Flow Cytometry Experiments

Stained cell mixtures were aliquoted in 1 mL volumes into 12×75 mm round bottom tubes. Inhibitors, such as latrunculin B, were added to both the experimental tube and a paired non-PTxM treated control tube 20 minutes prior to the PTxM treatment step. For experiments assessing temperature dependence, both the experimental tube and a paired non-PTxM treated control tube were placed at 37°C or on ice 20 minutes prior to the PTxM (or lectin) treatment. Experiments were performed in serum-free media to eliminate interactions of PTxM with fetuin, a protein found in fetal bovine serum. In order to eliminate possible differences due to serum starvation, PTxM, lectins, or dimers were added to the experimental tubes 30 min after the first serum-free media wash and incubated for 60 min unless otherwise specified in the text. Adherent cells (e.g. CHO) were treated at 37°C in 1.5 mL eppendorf tubes instead of 12×75 mm round bottom tubes and the incubation was performed on a rocker to minimize cellular adhesion to the tube walls. Following incubation with PTxM (or lectin), all cells were transferred to ice to halt further responses. Cells were then analyzed by flow cytometry on a FACSCalibur (Becton Dickinson, Mountain View, CA). All tubes were mixed well by pipetting immediately prior to analysis to break up loosely associated aggregates (unless otherwise indicated in text). Cells were flowed at 60 µL min^−1^ until 100,000 events were collected. Flow cytometry data were plotted and analyzed using FCS Express© software (De Novo Software, Los Angeles, CA). In order to determine the half maximal effective concentration (EC_50_), data was fitted to dose response models (GraphPad Software, La Jolla, CA). Each experimental condition was paired with an identical untreated control. Cell sorting was performed at Cincinnati Children’s Hospital Medical Center Research Flow Cytometry Core using a BD FACSAria II. Confocal studies were performed at the University of Cincinnati College of Medicine, Department of Molecular and Cellular Physiology, Live Microcopy Core, Zeiss LSM510 NLO Two-Photon Microscope, Microscope objectives: 40×/1.2 C-Apochromat (water immersion). Lasers for excitation of fluorescence: Ar (488 nm) and red HeNe (633 nm), Filters LP650 nm and BP 500–530 nm IR, transmitted light detector for laser-light Nomarski (differential interference contrast) imaging.

### Inositol Phosphate Assays

5×10^5^ Jurkat cells mL^−1^ were labeled with 1.5 µCi mL^−1^ [^3^H]myo-inositol (Perkin Elmer) for 18 hours in RMPI containing 10% serum. The cells were washed with cold PBS and suspended at 10^7^ cells mL^−1^ in serum-free RMPI containing 20 mM LiCl. 10^6^ cells/treatment were stimulated with PTx or PTx-S2/S4 dimers for 2 hours at 37°C. After stimulation, the cells were lysed with 0.4 M perchloric acid at 4°C for 15 minutes and neutralized with 0.72 M KOH and 0.6 M KHCO_3_. Total inositol phosphates were isolated with Dowex resin (BioRad), washed with water and eluted with 1 M ammonium formate/0.1 M formic acid. The eluted samples were counted with a liquid scintillation counter and the percent conversion of inositol phosphates from total incorporated [^3^H] was calculated. Data were graphed and analyzed using GraphPad Prism 4 software.

### Measurement of ERK Activity

1×10^6^ cells/treatment were serum starved for 1–2 hours and stimulated with PTx or PTx-S2/S4 dimers for 30 minutes at 37°C. After stimulation, cells were lysed directly in Laemmli sample buffer and analyzed for phosphorylated ERK 1 and 2 (phospho-ERK) and total ERK by western blotting.
